# New Insights into the Role of MHC Diversity in Devil Facial Tumour Disease

**DOI:** 10.1371/journal.pone.0036955

**Published:** 2012-06-06

**Authors:** Amanda Lane, Yuanyuan Cheng, Belinda Wright, Rodrigo Hamede, Laura Levan, Menna Jones, Beata Ujvari, Katherine Belov

**Affiliations:** 1 Faculty of Veterinary Science, University of Sydney, Sydney, Australia; 2 Department of Biological Sciences, Macquarie University, Sydney, Australia; 3 School of Zoology, University of Tasmania, Hobart, Australia; 4 School of Veterinary Medicine and Science, University of Nottingham, Nottingham, United Kingdom; Kyushu Institute of Technology, Japan

## Abstract

**Background:**

Devil facial tumour disease (DFTD) is a fatal contagious cancer that has decimated Tasmanian devil populations. The tumour has spread without invoking immune responses, possibly due to low levels of Major Histocompatibility Complex (MHC) diversity in Tasmanian devils. Animals from a region in north-western Tasmania have lower infection rates than those in the east of the state. This area is a genetic transition zone between sub-populations, with individuals from north-western Tasmania displaying greater diversity than eastern devils at MHC genes, primarily through MHC class I gene copy number variation. Here we test the hypothesis that animals that remain healthy and tumour free show predictable differences at MHC loci compared to animals that develop the disease.

**Methodology/Principal Findings:**

We compared MHC class I sequences in 29 healthy and 22 diseased Tasmanian devils from West Pencil Pine, a population in north-western Tasmania exhibiting reduced disease impacts of DFTD. Amplified alleles were assigned to four loci, *Saha-UA*, *Saha-UB*, *Saha-UC* and *Saha-UD* based on recently obtained genomic sequence data. Copy number variation (caused by a deletion) at *Saha-UA* was confirmed using a PCR assay. No association between the frequency of this deletion and disease status was identified. All individuals had alleles at *Saha-UD*, disproving theories of disease susceptibility relating to copy number variation at this locus. Genetic variation between the two sub-groups (healthy and diseased) was also compared using eight MHC-linked microsatellite markers. No significant differences were identified in allele frequency, however differences were noted in the genotype frequencies of two microsatellites located near non-antigen presenting genes within the MHC.

**Conclusions/Significance:**

We did not find predictable differences in MHC class I copy number variation to account for differences in susceptibility to DFTD. Genotypic data was equivocal but indentified genomic areas for further study.

## Introduction

The Tasmanian devil (*Sarcophilus harrisii*) is threatened with extinction in the wild due to the emergence of a contagious cancer known as Devil Facial Tumour Disease (DFTD). Since its emergence 15 years ago, devil populations have declined by at least 95% in the longest diseased areas and there are concerns that DFTD will lead to the extinction of this endangered marsupial carnivore within 35 years [1], [2]. DFTD is highly unusual in that it is transmitted as an infectious cell line (an allograft) [3]–[5] which spreads as devils bite and injure each other during the course of communal feeding on carcasses and during social and reproductive interactions [6], [7]. The primary DFTD tumours usually begin on the face or inside the mouth and quickly develop into large globular tumours, that ulcerate and become friable and metastasize to internal organs [4], [8]. DFTD is invariably fatal, usually within 3–6 months of the clinical presentation of visible tumours [9]. This has clear impacts, not just on devil population size and growth rates [10], but also age class structuring [11] and important life history traits including dispersal [12], sex ratios [11] and age at first reproduction [13].

One of the great challenges in managing this disease is that the cancer and its host are highly similar, since DFTD evolved from a Tasmanian devil Schwann cell (Schwann cells are part of the peripheral nervous system) [14]. Tasmanian devils are renowned for their lack of diversity across the genome [15], [16] including at a key gene region involved in immune response, the Major Histocompatibility Complex (MHC) [5]. The MHC plays a critical role in self/non-self recognition. MHC molecules can recognise antigenic peptides or cancer antigens and present them to cytotoxic T cells for destruction. MHC molecules can also act as antigens themselves, which is why MHC typing is used to select appropriate donors for tissue transplantation. Due to low levels of genetic diversity, devils frequently share the same MHC antigens as the DFTD tumour and hence it has been suggested that the tumour MHC antigens are not recognised by the devils as non-self [5]. Consequently, individual devils do not mount an immune response to DFTD [17]. We previously suggested that both the naturally existing contagious cancers, DFTD and Canine Transmissible Venereal Tumour (CTVT) evolved in populations of devils and wolves respectively that lacked MHC diversity [18]. It was postulated that these animals were more likely to be able to accept grafts from unrelated (but MHC identical) animals due to a lack of histocompatibility barriers. However, recent skin graft experiments have shown that MHC-similar devils will reject skin grafts [19]. This demonstrates that the low MHC diversity of devils does not necessarily mean that these animals are unable to identify foreign tissues. However, it does not address the possible influence that increased diversity at the MHC may have on reducing disease transmission.

Our research group previously surveyed sequence polymorphism in Tasmanian devils across their range and identified a total of 53 MHC class I sequence variants [20]. Between two and seven MHC class I variants were amplified from each individual, but without genomic information it was not possible to assign variants to loci. Recent sequencing of bacterial artificial chromosomes containing MHC inserts has allowed us to elucidate the gene content and organisation of the devil MHC. The Tasmanian devil MHC contains three classical class I genes (*Saha-UA*, *Saha-UB* and *Saha-UC*) [21]. These loci share extremely high amino acid identity in both exons (>98.3%) and introns (>97.7%) and account for all sequence variants which we previously designated “group 1” [20]. The sequences previously classified as “group 2” belong to a single locus designated *Saha-UD*, which shows some features of a nonclassical class I gene such as significantly lower levels of polymorphism [21]. Comparison of devil MHC haplotypes revealed a deletion within the *Saha-UA* gene, rendering it a pseudogene in certain haplotypes [21]. This deletion explains, to some extent, why a differing number of sequence variants have been detected between individuals.

It was previously suggested that individuals with a more restricted MHC complement (presence of only “group 1” or only “group 2” alleles) may be better able to recognize the foreign antigens on the surface of DFTD cells [20]. This is because the tumour (which contains genes which encode both group 1 and group 2 proteins) will therefore contain foreign MHC antigens which are not present in the host. We test this hypothesis by using individuals from a region of north-western Tasmania, West Pencil Pine (‘WPP’ hereafter). There is population genetic structuring across the island of Tasmania reflecting reduced gene flow between devils in the large, well connected eastern population and the more remote north-west region which is partially geographically isolated by large swathes of unsuitable alpine and wet forest habitat [22]. Devils in this region are genetically distinct from those in the east of Tasmania at both neutral loci [15],[16] and at MHC genes [20]. The devils at WPP are the first population to show reduced disease effects at both population and individual levels, with low disease prevalence, increased survival time of infected individuals, and little indication of changes in population size, population growth rate or age structure, four years after the disease was first detected [23]. Here we test the hypothesis that MHC class I differences can explain the variant epidemiology and host effects of DFTD at WPP. We do this by comparing the MHC profiles of devils that have presented with tumours with those of healthy older devils in the population (>3 years of age) who have not developed tumours. The rationale for selecting older individuals for the sample of uninfected devils was that they have had maximum time within the population to come into contact with DFTD without developing the disease. In eastern populations, virtually all devils have become infected and died of DFTD before the age of three [1], [10]. Data on contact rates and biting behavior of devils at WPP suggests that it is unlikely that mature adults that are mating and fighting would not have come into contact with DFTD by this age [6, R.H. unpublished data], particularly as the population is shown to act as one social unit [24].

We discover that mismatches in previously published polymerase chain reaction (PCR) primers result in inconsistent amplification of MHC alleles and turn to MHC-linked microsatellites to look for differences between the affected and unaffected groups, which proves to be an efficient alternative to cloning-and-sequencing based MHC typing in investigating genetic diversity and natural selection at MHC loci [25]–[27].

## Results and Discussion

### Tasmanian Devil MHC Class 1 Sequence

By establishing the presence/absence of alleles at the *Saha-UA* and *Saha-UD* loci via PCR assays, we detected no significant differences in MHC class I copy number between 22 diseased devils and 29 healthy controls from WPP. We propose that previous findings to the contrary were the result of inconsistent primer amplification. Sequencing of the MHC class I peptide binding region identified 27 different alleles including six variants previously undescribed. The six new alleles all clustered neatly within what was formerly referred to as the “group 1” clade of sequences (according to Siddle et al) [20] (*Saha-UA*, *UB* and *UC* loci according to Cheng et al) [21] and have been assigned GenBank accession numbers JN397396–JN397401.

Alleles were assigned to four loci (*Saha-UA, UB, UC* and *UD*) based on phylogenetic analysis (Table 1). Sequences associated with the *Saha-UD* locus were more divergent from the other loci, but overall sequence variation was still low and characterized by nucleotide identities of between 85–99% and amino acid identities of 74–100%. *Saha-UD* had the fewest alleles (four) and the highest within group nucleotide identities of 98–99% and amino acid identities of 96–100%. Locus *Saha-UB* had the largest number of alleles (ten) and the lowest within-group nucleotide identities of 96–99% and amino acid identities of 91–99%. *Saha-UA* and *UC* had six and seven alleles respectively and intermediate levels of nucleotide diversity. When alleles were translated into amino acids, all were non-synonymous with the exception of SahaI*32 and SahaI*39, which produced identical amino acid profiles (Table S1).

**Table 1:** Tasmanian devils sequenced using the MHC class I primers developed by Siddle et al [41] and screened for a deletion at the *Saha-UA* locus in this same gene region.

Disease status	Name	Deletion at*Saha-UA locus*	Alleles at*Saha-UA locus*		Alleles at*Saha-UB locus*		Alleles at*Saha-UC locus*		Alleles at*Saha-UD locus*		Missing data
Healthy	Allende	✓	SahaI*35		SahaI*49		SahaI*27				*Saha-UD*
	Baguette	χ	SahaI*29	SahaI*38			SahaI*27	SahaI*28	SahaI*32		
	Cartagena	χ	SahaI*35		SahaI*46	SahaI*49	SahaI*27	SahaI*96	SahaI*32		
	Chicomunita	χ	SahaI*35	SahaI*93	SahaI*46	SahaI*49	SahaI*27	SahaI*28			*Saha-UD*
	Chinquihue	χ			SahaI*36	SahaI*37					*Saha-UA, UC, UD*
	Delfina	χ	SahaI*29	SahaI*35			SahaI*27	SahaI*28			*Saha-UB, UD*
	Elvira	✓	SahaI*35		SahaI*49		SahaI*27		SahaI*32		
	Estrella	✓	SahaI*35		SahaI*49		SahaI*27				*Saha-UD*
	Evaristo	χ	SahaI*29				SahaI*27	SahaI*28	SahaI*32		*Saha-UB*
	Gengibre	χ			SahaI*48		SahaI*27	SahaI*53	SahaI*32		*Saha-UA*
	Huenchullan	✓	SahaI*35		SahaI*49		SahaI*27		SahaI*32		
	Iquique	χ	SahaI*35				SahaI*28		SahaI*39		*Saha-UB*
	Lascruces	✓					SahaI*27		SahaI*32		*Saha-UB*
	Lautaro	χ	SahaI*29	SahaI*35	SahaI*47		SahaI*27	SahaI*28			*Saha-UD*
	Limache	✓	SahaI*35		SahaI*49		SahaI*27	SahaI*91	SahaI*32		
	Mapuche	χ	SahaI*29	SahaI*35	SahaI*47		SahaI*27		SahaI*32		
	Melipilla	χ	SahaI*35		SahaI*49		SahaI*53	SahaI*96			*Saha-UD*
	Mirasol	χ			SahaI*37						*Saha-UA, UC, UD*
	Okapi	✓					SahaI*27		SahaI*32		*Saha-UB*
	Pomaire	✓	SahaI*29				SahaI*27	SahaI*28	SahaI*32		*Saha-UB*
	Puyehue	χ	SahaI*35		SahaI*49						*Saha-UC, UD*
	Racelette	✓			SahaI*36	SahaI*94	SahaI*27		SahaI*32		
	Sanantonio	✓	SahaI*35		SahaI*46		SahaI*28		SahaI*32		
	Sanvicente	χ	SahaI*29	SahaI*35			SahaI*28		SahaI*32		
	Segundo	χ	SahaI*29	SahaI*35	SahaI*49	SahaI*95	SahaI*27	SahaI*28	SahaI*32		
	Timoteo	✓			SahaI*36	SahaI*37	SahaI*27		SahaI*32		
	Trancura	✓	SahaI*35		SahaI*49		SahaI*27		SahaI*32	SahaI*75	
	Veronika	χ	SahaI*38		SahaI*49	SahaI*80	SahaI*27	SahaI*28	SahaI*32		
	Vieiochoco	χ	SahaI*35		SahaI*49		SahaI*27				*Saha-UD*
**Diseased**	Aconcagua	χ	SahaI*29	SahaI*35	SahaI*49		SahaI*27				*Saha-UD*
	Amor	χ	SahaI*35		SahaI*49		SahaI*27		SahaI*32		
	Arica	✓	SahaI*35				SahaI*28		SahaI*32		*Saha-UB*
	Calafquen	χ	SahaI*35	SahaI*92	SahaI*49		SahaI*27	SahaI*28	SahaI*32		
	Cassolette	✓	SahaI*35		SahaI*46		SahaI*28		SahaI*32	SahaI*67	
	Concon	✓					SahaI*27		SahaI*32		*Saha-UB*
	Copiapo	χ			SahaI*46	SahaI*49	SahaI*27	SahaI*28			*Saha-UA, UD*
	Curanto	✓	SahaI*35		SahaI*47						*Saha-UC, UD*
	Fabulosa	✓	SahaI*29		SahaI*80		SahaI*27	SahaI*28			*Saha-UD*
	Infima	✓			SahaI*49		SahaI*27	SahaI*28			*Saha-UD*
	Loncoche	✓	SahaI*35		SahaI*49	SahaI*95					*Saha-UC, UD*
	Malagente	✓	SahaI*29				SahaI*27	SahaI*28			*Saha-UB, UD*
	Marquez	✓					SahaI*27		SahaI*32		*Saha-UB*
	Mistral	✓	SahaI*29				SahaI*27	SahaI*28	SahaI*32		*Saha-UB*
	Negrita	χ	SahaI*29	SahaI*35	SahaI*49		SahaI*30				*Saha-UD*
	Olmue	χ	SahaI*35		SahaI*33		SahaI*28	SahaI*91			*Saha-UD*
	Princessa	χ	SahaI*35	SahaI*92	SahaI*49		SahaI*27	SahaI*28	SahaI*32		
	Renaca	✓	SahaI*35		SahaI*46		SahaI*28				*Saha-UD*
	Ruudgullit	χ	SahaI*29	SahaI*79	SahaI*36	SahaI*94	SahaI*27		SahaI*32		
	Saucisette	χ	SahaI*35	SahaI*92	SahaI*48	SahaI*49	SahaI*74				*Saha-UD*
	Tartiflette	χ	SahaI*29	SahaI*35	SahaI*49		SahaI*27	SahaI*28	SahaI*32		
	Tranamil	χ			SahaI*46		SahaI*28				*Saha-UA, UD*
	**Summary**	**45.01% deleted**	**9.80% missing**	**25.49% missing**	**9.80% missing**	**43.14% missing**					

In each individual devil the loci which can be confirmed as failing to amplify are identified (as indicated by no alleles present). No significant differences were found between healthy and diseased animals in the prevalence of the *Saha-UA* deletion or in the frequency with which alleles failed to amplify at any loci.

PCR primer efficiency was assessed for each locus, revealing the highest amplification failure rate of 43.14% at *Saha-UD* (Table 1). Locus specific primers for *Saha-UD* showed that all individuals had at least one allele and therefore previously reported copy number variation at this locus (‘group 2’) was the result of primer mismatches. Primer design represents one of the major technical challenges in MHC studies [28], [29]. In our case, the primer mismatches (two in *Saha-UA, UB* and *UC*, four in *Saha-UD*) were identified only after recent sequencing of BAC contigs for the devil MHC regions [21]. In the devil, copy number variation exists between different MHC haplotypes due to a deletion at the *Saha-UA* locus, making it more difficult to resolve whether differing numbers of alleles/genes are real or simply the result of primer inefficiency. Due to the high sequence similarity of *Saha-UA*, *Saha-UB* and *Saha-UC* it is impossible to develop locus specific primers for these loci. Previous sequencing of the devil MHC provided no evidence of a deletion at either *Saha-UB* or *Saha-UC* [21] and further work is underway to determine if copy number variations observed at these loci are real, or the result of poor sequence amplification. No significant differences were found between healthy and diseased devils in the frequency with which alleles failed to amplify at any of the four loci (*Saha-UA* p = 1.0; *Saha-UB* p = 0.76; *Saha-UC* p = 1.0; *Saha-UD* p = 0.17).

A specific PCR test was used to determine the frequency of copy number variation of *Saha-UA* as a result of a deletion at this locus. A deletion at *Saha-UA* occurred in 45.01% of all devils (Table 1). There was no significant difference between the two groups (healthy and diseased) in the frequency of this deletion (p = 0.58). It should be noted that the PCR test used to detect the deletion cannot resolve whether the deletion occurs in one or both haplotypes in the individual. Therefore, the association between missing one or two *Saha-UA* in a devil and the extent of DFTD susceptibility remains to be determined.

**Figure 1:**
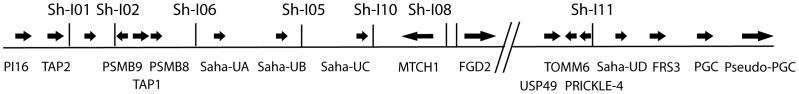
Location of eight MHC-linked microsatellites on devil chromosome four, associated with the MHC region. Six of these (Sh-I01, Sh-I02, Sh-I05, Sh-I06, Sh-I10 and Sh-I11) are located close to the four MHC class I loci (*Saha-UA*, *Saha-UB*, *Saha-UC* and *Saha-UD*) and several other genes involved in antigen presentation (TAP1, TAP2, PSMB8, PSMB9). The two remaining markers (Sh-I07, Sh-I08) are more closely linked with genes within the MHC that do not play a direct role in antigen presentation (MTCH1, FGD2).

Given the difficulties in differentiating real copy number variations from artifactual variations, we chose to employ MHC-linked microsatellite markers to assist in MHC typing healthy and diseased devils. These microsatellites are linked to various genes within the MHC region, including markers located close to each of the four MHC class I loci (*Saha-UA*, *Saha-UB*, *Saha-UC*, *Saha-UD*: Fig. 1) [30].

### Population Genetics Using Neutral and MHC-linked Microsatellite Markers

Nine neutral microsatellite markers provided a background for assessing selection on the MHC [31]. All neutral markers conform to Hardy-Weinberg expectations and display similar numbers of alleles, allele frequencies and levels of heterozygosity for diseased and healthy animals (Table 2). Population pairwise F_ST_ = 0.005 (p = 0.21±0.004) indicates no significant difference between diseased and healthy animals at these loci. As no demographic factors such as migration or assortative mating are evident in the neutral markers, any deviations from Hardy-Weinberg equilibrium observed at MHC-linked loci may be attributed to selection.

**Table 2:** Neutral and MHC-linked microsatellite loci summary statistics for diseased (shaded rows) and healthy devils.

Neutral loci					MHC-linked loci				
Locus	FIS	A	Ho/He	HWE p value (±s.d.)	Locus	FIS	A	Ho/He	HWE p value (±s.d.)
Sh2i	0.215	3	0.44/0.56	0.388 (±0.001)	ShI01	−0.240	3	0.82/0.67	0.333 (±0.001)
	0.274	3	0.28/0.38	0.059 (±0.001)		−0.018	3	0.68/0.67	0.134 (±0.001)
Sh2g	−0.048	3	0.50/0.48	0.723 (±0.001)	ShI02	0.051	2	0.41/0.43	1.000 (±0.000)
	0.091	3	0.45/0.50	0.137 (±0.001)		0.171	2	0.39/0.47	0.435 (±0.002)
Sh2v	0.046	5	0.68/0.71	0.111 (±0.001)	ShI05	0.102	2	0.38/0.42	0.660 (±0.002)
	0.017	4	0.67/0.68	0.916 (±0.001)		0.277	2	0.32/0.44	0.209 (±0.001)
Sh5c	−0.099	3	0.59/0.54	0.893 (±0.001)	ShI06	−0.051	2	0.48/0.46	1.000 (±0.000)
	0.194	3	0.40/0.49	0.323 (±0.001)		0.030	2	0.45/0.47	1.000 (±0.000)
Sh6e	0.010	2	0.33/0.34	1.000 (±0.000)	ShI07	−0.107	5	0.79/0.71	0.753 (±0.001)
	−0.191	2	0.34/0.29	0.556 (0.001)		−0.092	5	0.76/0.70	**0.003 (±0.000)** *
Sh6L	0.315	2	0.25/0.36	0.119 (±0.001)	ShI08	−0.053	6	0.76/0.72	0.940 (±0.001)
	0.142	2	0.36/0.42	0.640 (±0.002)		−0.040	6	0.81/0.78	0.169 (±0.001)
Sh2L	−0.059	2	0.14/0.14	1.000 (±0.000)	ShI10	0.097	3	0.45/0.50	0.465 (±0.002)
	−0.019	2	0.07/0.07	1.000 (±0.000)		−0.120	4	0.31/0.28	1.000 (±0.000)
Sh2p	−0.104	4	0.68/0.62	0.889 (±0.001)	ShI11	−0.007	4	0.64/0.64	0.465 (±0.002)
	−0.014	3	0.46/0.46	1.000 (±0.000)		0.290	3	0.45/0.63	0.153 (±0.001)
Sh3a	−0.027	2	0.44/0.43	1.000 (±0.000)					
	0.006	2	0.48/0.48	1.000 (±0.000)					

A single locus (Sh-I07) displays a departure from Hardy-Weinberg equilibrium for healthy devils only. Numbers of alleles and levels of heterozygosity are very similar for healthy and DFTD infected devils though with a slight trend for higher heterozygosity in infected devils.

* denotes statically significant departure from Hardy-Weinberg equilibrium (HWE). FIS indicates homozygote (+ve) or heterozygote (−ve) excess. A is the number of alleles. Ho/He is observed and expected heterozygosities.

**Figure 2:**
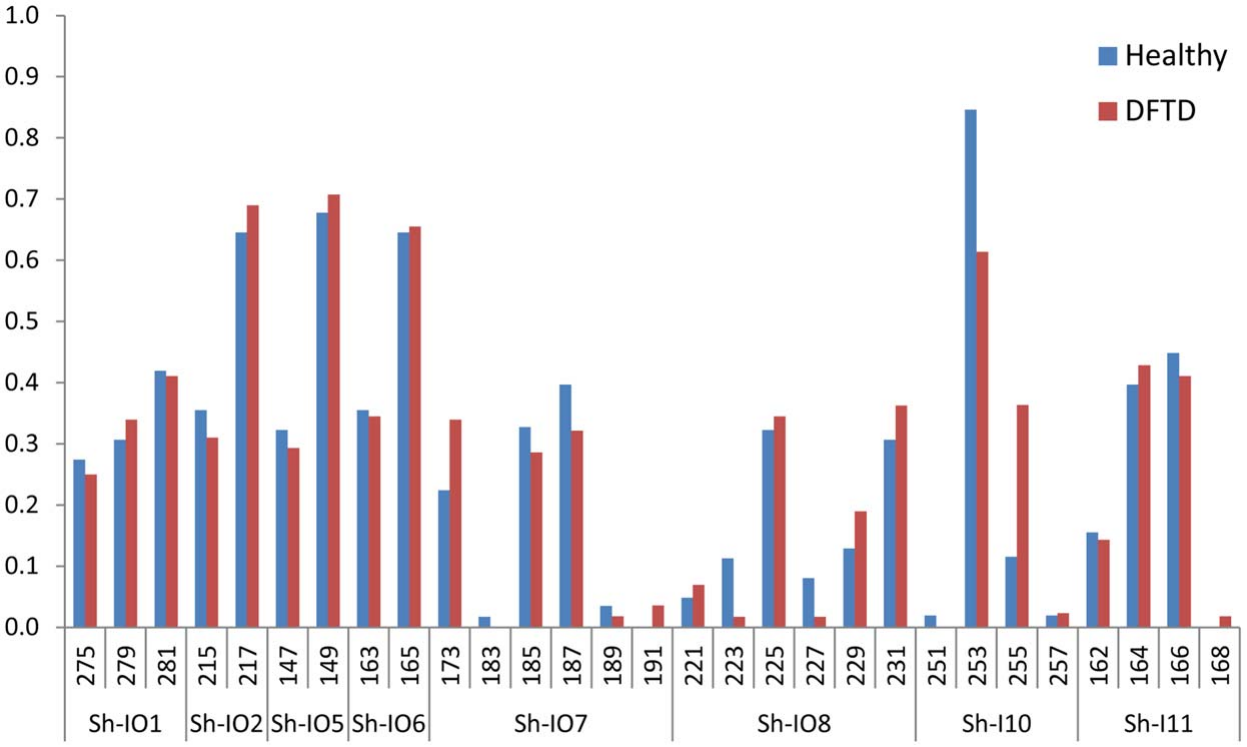
MHC-linked microsatellite loci allele frequencies showing little variation between healthy and DFTD infected devils. A single locus (Sh-I07) does not conform to Hardy-Weinberg expectations for healthy devils only (p = 0.003).

The eight MHC-linked microsatellite markers also display similar levels of heterozygosity, allele frequencies and identical numbers of alleles for diseased and healthy devils (Fig. 2, Table 2). Linkage disequilibrium was high for almost all MHC-linked loci, which is expected as they all map to chromosome 4 q [30] (Fig. 1). While heterozygosity was relatively high for some loci (Sh-I01, Sh-I07, Sh-I08), Hardy-Weinberg expectations held for all but one locus (Sh-I07) for healthy devils only (p = 0.003, Table 2). This remained significant after sequential Bonferroni correction. In sum, these results suggest no strong heterozygote advantage nor the presence of DFTD-resistance alleles. Further, population pairwise F_ST_ = 0.0007 (p = 0.41±0.006) indicates no differentiation between diseased and healthy devils.

**Figure 3:**
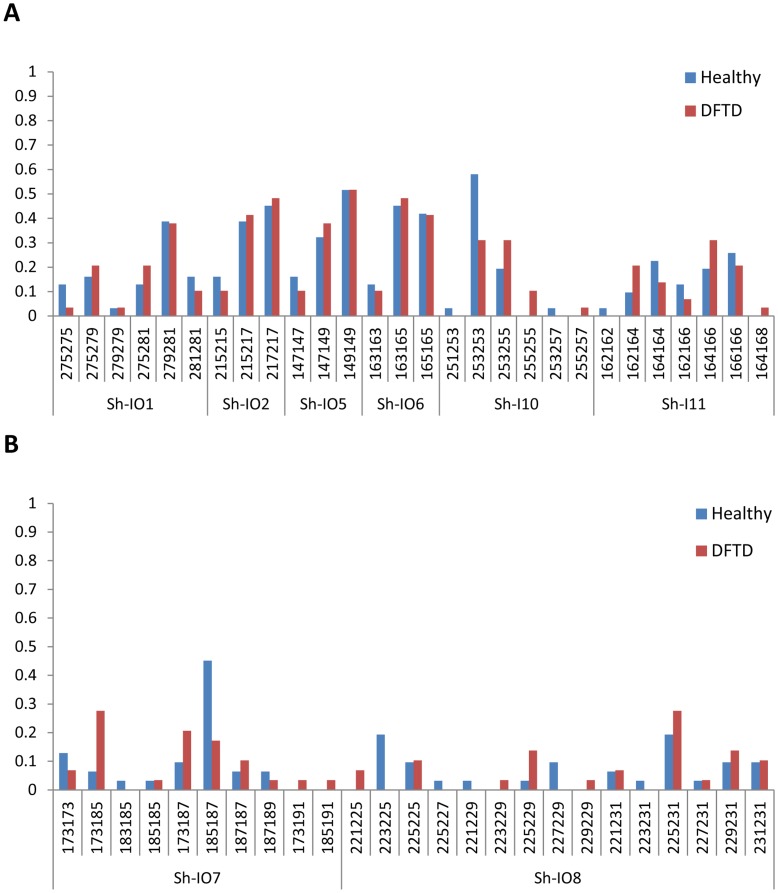
Genotype frequencies for healthy and DFTD infected devils. (**A**) At the six microsatellite loci associated with antigen-presenting genes within the MHC (Sh-I01, Sh-I02, Sh-I05, Sh-I06, Sh-I10 and Sh-I11). No deviations from Hardy-Weinberg equilibrium are observed for either healthy or DFTD infected devils. (**B**) For two microsatellite markers more closely associated with non-antigen presenting genes within the MHC region (Sh-I07 and Sh-I08). The Sh-I07 locus is out of Hardy-Weinberg equilibrium for healthy devils only (p = 0.029) and the Sh-I08 locus does not conform to Hardy-Weinberg equilibrium at the 0.1 significance level (p = 0.076). Three differences in genotype frequencies were significant before Bonferroni correction (Sh-I07∶173/185, p = 0.041 and 185/187, p = 0.024; Sh-I08∶223/225, p = 0.024).

The frequency of genotypes in the population was also assessed. This measure is particularly sensitive to changes over short time scales (allele frequencies take longer to change in a population than genotype frequencies), which is relevant considering the recent arrival of DFTD in the region. Microsatellite markers located closest to antigen presenting MHC class I loci (Sh-I01, Sh-I02, Sh-I05, Sh-I06, Sh-I10, Sh-I11) had low allelic (Fig. 2) and genotype diversity (Fig. 3a), possibly the result of a selective sweep due to a prior disease epidemic [32], [33]. Two markers (Sh-I07 and Sh-I08) are located within the MHC but are closest to non-antigen presenting genes, MTCH1 and FGD2 (Fig. 3). Both loci have considerably higher polymorphism with six alleles each and 10–15 genotypes (mean 12.5), compared to 2–4 alleles (mean 2.83) and 3–7 genotypes (mean 4.67) in the six remaining markers which are located closest to genes involved in antigen presentation.

Monte Carlo analysis of genotype distributions showed that the actual distribution of genotypes, when compared across the two subgroups (healthy and diseased), differed significantly from 10,000 randomly generated distributions for one locus (Sh-I07 p = 0.04). Fishers exact test was then used to investigate in more detail which particular genotypes differed in frequency between healthy and diseased devils. Three differences were identified in genotype frequency at two separate loci, which were significant before sequential Bonferroni correction (Sh-I07 genotype 173/185, p = 0.041 and 185/187, p = 0.024; Sh-I08 genotype 223/225, p = 0.024: Fig. 3b), though not afterwards due to the large number of comparisons. These differences warrant further investigation, and future work will focus on increasing the number of markers in these regions.

As mentioned above, both Sh-I07 and Sh-I08 are located within the MHC, but are physically located closer to non-antigen presenting genes than to antigen presenting genes. Sh-108 is located 14.4 kb upstream of MTCH1 (also referred to as PSAP), an evolutionarily conserved gene important in mitochondrial transport [34]. Interestingly, this gene plays a key role in apoptosis – a form of programmed cell death which is crucial in maintaining health as it eliminates old and unhealthy cells [35]–[37]. Sh-I07 is found 5.5 kb away from FGD2, a gene primarily responsible for the formation of microskeletal structures [38]. This gene also plays a role in immunity via leukocyte signaling and it is known to be expressed in B lymphocytes, macrophages and dendritic cells [39]. In all cases it is possible that the signals we are detecting are simply due to a hitchhiking effect and that use of additional markers within the region may help pinpoint stronger associations.

### Conclusions and Implications for Tasmanian Devil Management

The reduced effects of DFTD at the north-western site of WPP, including low disease prevalence and no population decline [23] are not due to copy number variation of MHC class I ‘group 2’ (*Saha-UD*) alleles, as previously proposed [20]. We show that perceived differences at this locus are due to inconsistent primer amplification as all individuals are confirmed to have alleles present. No differences are detected between healthy and diseased devils in either the frequency of MHC-linked microsatellite alleles (indicating no ‘resistance/susceptibility’ alleles) or in the prevalence of a deletion of locus *Saha-UA*, signifying no influence of copy number variation at this locus. The results of genotype frequency analysis are equivocal with some evidence that certain genotypes are more frequently encountered in healthy devils. As the significance of differences in individual genotype frequencies disappeared after Bonferroni correction, we cannot rule out the possibility that these differences are due to chance, though they do present these genes as being of interest for further study. Future work should aim to increase sample sizes and the number of markers in the region to determine whether these results are real or the result of chance alone.

The different epidemiology of DFTD in WPP is currently unexplained. The possibility remains that the uninfected devils used in our analysis may have the disease but not yet developed tumours or that they have simply not yet come into contact with the disease. Both of these explanations are plausible but unlikely. The latency state, between infection and the presentation of tumours, is estimated to average 6 months but could be at least 10 months [1, R.H. unpublished data]. Low disease prevalence among adults (13%) observed in WPP at the time of this study, relative to a large devil population (300 individuals trapped between 2006 and 2011), means that the number of ‘latent’ animals would be low. Older devils greater than three years of age were specifically selected for our analysis as they have had maximum opportunity for exposure to DFTD including social and reproductive interactions which involve biting. By three years of age virtually all devils in eastern Tasmania have succumbed to the disease and the remainder die within months [1], [10]. While there is no current evidence that animals at WPP differ from those in the east in behaviours linked to disease transmission, we suggest modeling the disease risks of each population as a research priority.WPP provides our best hopes of determining whether genetic resistance to DFTD exists. Future studies will continue to target the MHC, as well as expanding to look genome-wide, using techniques such as SNPs (single nucleotide polymorphisms) and next generation sequencing to identify the impact that immune gene variants have on disease susceptibility.

## Methods

### Ethics Statement

The field research was carried out with approval from the University of Tasmania’s Animal Ethics Committee (A0010296) and from the Tasmanian Department of Primary Industries and Water (TFA 08211).

### Study Site and Sample Collection

West Pencil Pine (41° 31′ S, 145° 46′ E) is a 25 km^2^ area situated on private production forestry land to the west of Cradle Mountain in northwest Tasmania. This study site was established in May 2006 (when the disease was first detected in the region), following three exploratory expeditions aimed at locating the epidemic front. We subsequently sampled this population four times per year, from August 2006 until May 2010, at three month intervals. Forty carnivore traps were set for 10 nights each trip. All individuals caught were individually marked using implantable microchip transponders and a 3 mm biopsy of ear tissue was taken using a sterile biopsy punch. Due to the regularity with which this site has been monitored, most of the individuals in our data set were originally captured as sub-adults and are therefore of known age. We aged devils first captured as adults using a combination of molar eruption, molar tooth wear and canine over eruption. This method is considered precise for ageing devils up to three years of age (M.J., unpublished data). Disease status was assessed by histopathological examination of biopsies from tumours [8], or when this was not possible, by visual inspection and identification of tumours [9].

The healthy and diseased devils were trapped and genetically sampled between May 2006 and May 2010. All the healthy, tumour-free devils used in this study were greater than three years old at the time of capture and had co-existed with DFTD infected individuals at least since sexual maturity for males, and for most females, for their entire lives (devils have male-biased natal dispersal) [12].

### DNA Extraction and MHC Class I Sequencing

Total genomic DNA was extracted from ear biopsies using the Hot-SHOT extraction method [40]. A fragment that targeted the MHC class I α 1 domains (exon 2), was amplified in polymerase chain reactions (PCR) utilizing primers developed by Siddle et al. [41]. Amplification reactions were 25 µL volume and comprised; 1 x High Fidelity Buffer (Invitrogen) consisting of 60 mM Tris-HCl (pH 8.9) and 18 mM (NH_4_)_2_SO_4_, 2 mM MgSO_4_, 0.2 mM dNTPs, 0.5 µM forward and reverse primers, 1.5 U of Platinum *Taq* DNA Polymerase High Fidelity (Invitrogen) and approximately 10 ng of template DNA. PCR amplification of double-stranded product was performed with a MJ Mini Personal Thermocycler (Biorad) using a cycling profile consisting of an initial denaturing step of 94C for 3 mins followed by 35 cycles of 94C for 30 s, 60C for 30 s and 72C for 30 s. This was followed by a final extension at 72C for 20 mins. PCR products were checked for quality on a 1% agarose gel and DNA concentration measured using a Nano Photometer (Implen). Bands of approximately 300 bp in size were extracted from agarose gels using an Ultra Clean DNA Purification Kit (MoBio) and cloned into pGEM-T easy vector (Promega) according to the manufacturer’s protocol. Colonies with inserts of the predicted size were inoculated into LB broth, grown overnight, and DNA was extracted using a QIAprep spin miniprep kit (Qiagen). In total 10 clones each from two separate PCRs were sequenced per individual, resulting in a total of 1060 sequences (sequencing performed by Australian Genome Research Facility, Queensland).

Sequences were aligned and quality-checked using Bioedit v7.0.9 [42]. All sequences containing ambiguous nucleotides were removed from analysis. To confirm the validity of new sequence variants and establish that they were not the result of PCR error, new sequences had to occur in a minimum of two separate PCR amplifications (ie., either two separate PCRs of the same individual or PCRs of two different devils). All new sequences were translated into amino acids using the standard genetic code and checked for the presence of stop codons in MEGA v 5 [43]. Novel nucleotide sequences were submitted to Genbank. They were placed in the context of other, previously identified devil MHC sequences via phylogenetic analysis. MEGA v 5 [43] was used to build a neighbor joining tree using Jukes-Cantor distance with 1000 bootstraps, consistent with the methods used to build the phylogeny of marsupial class I sequences [44].

Two PCR tests were performed to confirm the existence of *Saha-UA* and *Saha-UD* in each individual. The copy number variation at *Saha-UA* was detected following the protocol described previously [21]. A new pair of primers (forward 5′- ATGGATAGAGAAGATGGAGAAT -3′ and reverse 5′- CTGGTTGTAGTAGCCGTGTA -3′) were designed to specifically amplify a 121 bp segment within the α1 domain of *Saha-UD* at the following conditions: 94°C initial denaturation for 3 min; 32 cycles of 94°C denaturation for 30 s, 56°C annealing for 30 s, 72°C extension for 30 s; and 72°C final extension for 10 min.

### Microsatellite Genotyping

Eight molecular markers linked to MHC-loci [30] were used to genotype 29 diseased and 31 healthy Tasmanian devils. Note that sample number includes seven additional diseased animals than were used in sequencing and two additional healthy animals. Other than the inclusion of these extra nine devils, the same individuals were used for both sequencing and microsatellite analyses. Microsatellite loci were amplified in 15 µL reactions using approximately 10 ng of genomic DNA with 0.2 mM dNTPs, 1 x PCR buffer (containing 15 mM MgCl_2_), additional 2 mM MgCl_2_, 0.5 mM unlabelled primer, 0.05 mM primer labeled with M13 (−21) tail, 0.5 mM universal fluorescent-labelled M13(−21) primer, 25 ng/µL BSA (Bovine Albumin Serum) and 0.05 U of *Taq* DNA polymerase (Qiagen). Forward and reverse primers were labeled as in Cheng et al [30]. PCR reactions were performed with a MJ Mini Personal Thermocycler (Biorad) under the following conditions: 94°C for 3 mins, 6 cycles of 94°C for 30 s, 60°C for 30 s and 72°C for 30 s using a touchdown program with the annealing temperature decreasing by 1°C per cycle for a final temperature of 54°C; 30 cycles of 94°C for 30 s, 54°C for 30 s, 72°C for 30 s and a final extension of 72°C for 10 mins.

Nine neutral microsatellite markers [45] were also used to genotype the same 29 DFTD-infected and 31 healthy animals. Neutral loci were amplified in 15 µL reactions using approximately 10 ng of genomic DNA, 0.2 mM dNTPs, 1 x PCR buffer (containing 15 mM MgCl_2_), additional 0.5 mM MgCl_2_, 0.2 mM forward and reverse primers labeled as in Jones et al [45] (fluorescent labels: Applied Biosytems) 0.025 U of *Taq* DNA polymerase (Qiagen). Reactions were performed with a MJ Mini Personal Thermocycler (Biorad) under the following conditions: 94°C for 1.45 mins, 6 cycles of 94°C for 15 s, 60°C for 20 s and 72°C for 15 s using a touchdown program with the annealing temperature decreasing by 1°C per cycle for a final temperature of 54°C; 30 cycles of 94°C for 15 s, 54°C for 20 s, 72°C for 10 s and final extension of 72°C for 2 mins.

The amplified products were separated by electrophoresis on an ABI 3130XL sequencer (Applied Biosystems) and scored against the size marker LIZ 500 using Genemarker v 1.95 (Soft Genetics LLC).

### Analysis

The frequency of a deletion at the *Saha-UA* locus was compared between healthy and diseased devils using two-tailed Fisher’s Exact Test in SPSS version 18 (SPSS Inc). This same method was used to compare the likelihood of the primers failing to amplify alleles a given locus in either healthy or diseased animals. Cases where only one allele was detected may be the result of a failure to amplify, but conversely the individual may be homozygous. For this reason only when a locus was missing all alleles did we consider this conclusive evidence of non-amplification.

Population genetic (microsatellite) analysis was performed using Arlequin: version 3.11 [46] and FSTAT: version 2.9.3.2 [47]. Hardy-Weinberg equilibrium was calculated in Arlequin via an exact test using a Markov chain (forecasted chain length: 100,000, dememorisation steps: 5000) [48]. Population structure was analysed using Arlequin to calculate F_ST_ values (distance method, 10,100 permutations) as well as F_IS_ for each category- diseased and healthy. FSTAT was used to calculate linkage disequilibrium. Allele and genotype frequencies were calculated using GenAlEx [49] and compared between subgroups using two-tailed Fishers Exact Test in SPSS version 18 (SPSS Inc). Allelic diversity and observed and expected heterozygosities were calculated with Arlequin and results were corrected by sequential Bonferroni analysis [50]. A Monte Carlo test of randomization was used to assess whether the observed distribution of genotypes at each locus differed significantly from 10,000 randomly generated distributions. Compared to parametric tests, Monte Carlo procedures are more robust to small and unbalanced datasets [51]. The program PopTools v 3.2 [52] provided the shuffle algorithm for generating random distributions as well as the difference statistic used for the Monte Carlo simulation. P values were calculated according to the number of times the actual distribution of genotypes across the two disease statuses (diseased and non-diseased) fell outside the confidence limits (lower percentile = 0.025, upper percentile = 0.975) of 10,000 randomly generated distributions.

## Supporting Information

Table S1 **Amino acid sequences of all Tasmanian devil MHC class I alleles amplified in this study.** The consensus sequence is SahaI*27 and all polymorphic residues are shown.
(DOCX)
