# Supplementary material for: New Insights into the Role of MHC Diversity in Devil Facial Tumour Disease
Source: PLoS One. 2012 Jun 6;7(6):e36955. doi: 10.1371/journal.pone.0036955 (PMC3368896; doi:10.1371/journal.pone.0036955)
Supplement: Table S1 — Amino acid sequences of all Tasmanian devil MHC class I alleles amplified in this study. The consensus sequence is SahaI*27 and all polymorphic residues are shown. (DOCX) [file pone.0036955.s001.docx]

**Supplementary Table 1**: Amino acid sequences of all Tasmanian devil MHC class I alleles amplified in this study. The consensus sequence is SahaI*27 and all polymorphic residues are shown.

|  | 1 |  |  |  |  |  |  |  |  | 20 | |  |  |  |  |  |  |  |  | 20 | |  |  |  |  |  |  |  |  | 30 | |  |  |  |  |  |  |  |  | 40 | |  |  |
| --- | --- | --- | --- | --- | --- | --- | --- | --- | --- | --- | --- | --- | --- | --- | --- | --- | --- | --- | --- | --- | --- | --- | --- | --- | --- | --- | --- | --- | --- | --- | --- | --- | --- | --- | --- | --- | --- | --- | --- | --- | --- | --- | --- |
| Saha-27 | T | A | V | S | R | P | G | L | G | E | P | R | F | L | A | V | G | Y | V | D | D | Q | Q | F | V | R | F | D | S | D | S | A | S | Q | S | E | E | P | R | A | P | W | M |
| Saha-28 |  |  |  |  |  |  |  |  |  |  |  |  |  |  | S |  |  |  |  |  |  |  |  |  |  |  |  |  |  |  |  |  |  |  |  |  |  |  |  |  |  |  |  |
| Saha-29 |  |  |  |  |  |  |  |  |  |  |  |  |  |  | S |  |  |  |  |  |  |  |  |  |  |  |  |  |  |  |  |  |  |  |  |  |  |  |  |  |  |  |  |
| Saha-30 |  |  |  |  |  |  |  |  |  |  |  |  |  |  | S |  |  |  |  |  |  |  |  |  |  |  |  |  |  |  |  |  |  |  |  |  |  |  |  |  |  |  | I |
| Saha-32 |  | T |  |  |  |  |  |  |  |  |  |  |  | F | S |  |  |  |  |  |  |  |  |  |  | G |  |  |  |  |  |  |  |  | R | V |  |  |  |  |  |  | I |
| Saha-33 |  |  |  |  |  |  |  |  |  |  |  |  |  |  | S |  |  |  |  |  |  |  |  |  |  |  |  |  |  |  |  |  |  |  |  |  |  |  |  |  |  |  |  |
| Saha-35 |  |  |  |  |  |  |  |  |  |  |  |  |  |  | S |  |  |  |  |  |  |  |  |  |  |  |  |  |  |  |  |  |  |  |  |  |  |  |  |  |  |  |  |
| Saha-36 |  |  |  |  |  |  |  |  |  |  |  |  |  |  | S |  |  |  |  |  |  |  |  |  |  |  |  |  |  |  |  |  |  |  |  |  |  |  |  |  |  |  |  |
| Saha-37 |  |  |  |  |  |  |  |  |  |  |  |  |  |  |  |  |  |  |  |  |  |  |  |  |  |  |  |  |  |  |  |  |  |  |  |  |  |  |  |  |  |  |  |
| Saha-38 |  |  |  |  |  |  |  |  |  |  |  |  |  |  |  |  |  |  |  |  |  |  |  |  |  |  |  |  |  |  |  |  |  |  |  |  |  |  |  |  |  |  |  |
| Saha-39 |  | T |  |  |  |  |  |  |  |  |  |  |  | F | S |  |  |  |  |  |  |  |  |  |  | G |  |  |  |  |  |  |  |  | R | V |  |  |  |  |  |  | I |
| Saha-46 |  |  |  |  |  |  |  |  |  |  |  |  |  |  |  |  |  |  |  |  |  |  |  |  |  |  |  |  |  |  |  |  |  |  |  |  |  |  |  |  |  |  |  |
| Saha-47 |  |  |  |  |  |  |  |  |  |  |  |  |  |  |  |  |  |  |  |  |  |  |  |  |  |  |  |  |  |  |  |  |  |  |  |  |  |  |  |  |  |  |  |
| Saha-48 |  |  |  |  |  |  |  |  |  |  |  |  |  |  |  |  |  |  |  |  |  |  |  |  |  |  |  |  |  |  |  |  |  |  |  |  |  |  |  |  |  |  |  |
| Saha-49 |  |  |  |  |  |  |  |  |  |  |  |  |  |  |  |  |  |  |  |  |  |  |  |  |  |  |  |  |  |  |  |  |  |  |  |  |  |  |  |  |  |  |  |
| Saha-53 |  |  |  |  |  |  |  |  |  |  |  |  |  |  | S |  |  |  |  |  |  |  |  |  |  |  |  |  |  |  |  |  |  |  |  |  |  |  |  |  |  |  |  |
| Saha-67 |  |  |  |  |  |  |  |  |  |  |  |  |  |  | S |  |  |  |  |  |  |  |  |  |  | G |  |  |  |  |  |  |  |  | R | V |  |  |  |  |  |  | I |
| Saha-74 |  |  |  |  |  |  |  |  |  |  |  |  |  |  |  |  |  |  |  |  |  |  |  |  |  |  |  |  |  |  |  |  |  |  |  |  |  |  |  |  |  |  |  |
| Saha-75 |  | T |  |  |  |  |  |  |  |  |  |  |  | F | S |  |  |  |  |  |  |  |  |  |  | G |  | N |  |  |  |  |  |  | R | V |  |  |  |  |  |  | I |
| Saha-79 |  |  |  |  |  |  |  |  |  |  |  |  |  |  | S |  |  |  |  |  |  |  |  |  |  |  |  |  |  |  |  |  |  |  |  |  |  |  |  |  |  |  |  |
| Saha-80 |  |  |  |  |  |  |  |  |  |  |  |  |  |  | S |  |  |  |  |  |  |  |  |  |  |  |  |  |  |  |  |  |  |  |  |  |  |  | Q |  |  |  |  |
| Saha-91 |  |  |  |  |  |  |  |  |  |  |  |  |  |  | T |  |  |  |  |  |  |  |  |  |  |  |  |  |  |  |  |  |  |  |  |  |  |  |  |  |  |  |  |
| Saha-92 |  |  |  |  |  |  |  |  |  |  |  |  |  |  |  |  |  |  |  |  |  |  |  |  |  |  |  |  |  |  |  |  |  |  |  |  |  |  |  |  |  |  |  |
| Saha-93 |  |  |  |  |  |  | R |  |  |  |  |  |  |  | S |  |  |  |  |  |  |  |  |  | M | |  |  |  |  |  |  |  |  |  |  |  |  |  |  |  |  |  |
| Saha-94 |  |  |  |  |  |  |  |  |  |  |  |  |  |  |  |  |  |  |  |  |  |  |  |  |  |  |  |  |  |  |  |  |  |  |  |  |  |  |  |  |  |  |  |
| Saha-95 |  |  |  |  |  |  |  |  |  |  |  |  |  |  | S |  |  |  |  |  |  |  |  |  |  |  |  |  |  |  |  |  |  |  |  |  |  |  |  |  |  |  |  |
| Saha-96 |  |  |  |  |  |  |  |  |  |  |  |  |  |  | S |  |  |  |  |  |  |  |  |  |  |  |  |  |  |  |  |  |  |  |  |  |  |  |  |  |  |  |  |

|  |  |  |  |  |  |  | 50 | |  |  |  |  |  |  |  |  | 60 | |  |  |  |  |  |  |  |  | 70 | |  |  |  |  |  |  |  |  | 80 | |  |
| --- | --- | --- | --- | --- | --- | --- | --- | --- | --- | --- | --- | --- | --- | --- | --- | --- | --- | --- | --- | --- | --- | --- | --- | --- | --- | --- | --- | --- | --- | --- | --- | --- | --- | --- | --- | --- | --- | --- | --- |
| Saha-27 | E | K | V | Q | D | V | D | P | G | Y | W | E | R | N | T | Q | I | S | K | E | N | A | Q | S | S | R | V | S | L | Q | N | L | R | G | Y | F | N | Q | S |
| Saha-28 |  |  |  |  |  |  |  |  |  |  |  |  |  |  |  |  |  |  |  |  |  |  |  |  |  |  |  |  |  |  |  |  |  |  |  |  |  |  |  |
| Saha-29 |  |  |  |  |  |  |  |  |  |  |  |  | Q | E |  |  |  | I |  |  | T |  |  | I |  |  |  | D |  |  | T |  |  |  |  |  |  |  |  |
| Saha-30 |  |  |  |  |  |  |  |  |  |  |  |  |  |  |  |  |  |  |  |  |  |  |  |  |  |  |  |  |  |  |  |  |  |  |  |  |  |  |  |
| Saha-32 |  |  | M | E | N |  |  | R | D |  |  |  |  |  |  |  | N |  |  | R |  |  |  | I |  |  | E | D |  |  | T |  | H |  |  |  |  |  |  |
| Saha-33 |  |  |  | K |  |  |  |  |  |  |  |  | Q | Q |  |  |  | I |  |  | T |  |  | I | Y |  |  | G |  |  | T |  |  |  |  |  |  |  |  |
| Saha-35 |  |  |  | K |  |  |  |  |  |  |  |  | Q | E |  |  |  | I |  |  | T |  |  | I |  |  |  | D |  |  | T |  |  |  |  |  |  |  |  |
| Saha-36 |  |  |  | K |  |  |  |  |  |  |  |  | Q | E |  |  |  |  |  |  | T |  |  | I | Y |  |  | G |  |  | T |  |  |  |  |  |  |  |  |
| Saha-37 |  |  |  | K |  |  |  |  |  |  |  |  | Q | E |  |  |  |  |  |  |  |  |  | I | Y |  |  | G |  |  | T |  |  |  |  |  |  |  |  |
| Saha-38 |  |  |  |  |  |  |  |  |  |  |  |  | Q | E |  |  |  | I |  |  | T |  |  | I |  |  |  | D |  |  | T |  |  |  |  |  |  |  |  |
| Saha-39 |  |  | M | E | N |  |  | R | D |  |  |  |  |  |  |  | N |  |  | R |  |  |  | I |  |  | E | D |  |  | T |  | H |  |  |  |  |  |  |
| Saha-46 |  |  |  |  |  |  |  |  |  |  |  |  | Q | E |  |  |  | I |  |  |  |  |  |  |  |  |  | D |  |  | T |  |  |  |  |  |  |  |  |
| Saha-47 |  |  |  |  |  |  |  |  |  |  |  |  | Q | Q |  |  | N |  |  | G |  |  |  | I | Y |  |  | G |  |  | T |  |  |  |  |  |  |  |  |
| Saha-48 |  |  |  |  |  |  |  |  |  |  |  |  | Q | E |  |  |  | I |  |  |  |  |  |  |  |  |  |  |  |  |  |  |  |  |  |  |  |  |  |
| Saha-49 |  |  |  | K |  |  |  |  |  |  |  |  | Q | Q |  |  |  |  |  |  |  |  |  | I | Y |  |  | G |  |  | T |  |  |  |  |  |  |  |  |
| Saha-53 |  |  |  | K |  |  |  |  |  |  |  |  |  |  |  |  |  |  |  |  |  |  |  |  |  |  |  |  |  |  | T |  |  |  |  |  |  |  |  |
| Saha-67 |  |  | M | E | N |  |  | R | D |  |  |  |  |  |  |  | N |  |  | R |  |  |  | I |  |  | E | D |  |  | T |  | H |  |  |  |  |  |  |
| Saha-74 |  |  |  |  |  |  |  |  |  |  |  |  |  |  |  |  |  |  |  |  |  |  |  |  |  |  |  |  |  |  | T |  |  |  |  |  |  |  |  |
| Saha-75 |  |  | M | E | N |  |  | R | D |  |  |  |  |  |  |  | N |  |  | R |  |  |  | I |  |  | E | D |  |  | T |  | H |  |  |  |  |  |  |
| Saha-79 |  |  |  | K |  |  |  |  |  |  |  |  | Q | E |  |  |  |  |  |  |  |  |  | I | Y |  |  | G |  |  | T |  |  |  |  |  |  |  |  |
| Saha-80 |  |  |  | K |  |  |  |  |  |  |  |  | Q | E |  |  |  |  |  |  |  |  |  |  |  |  |  |  |  |  |  |  |  |  |  |  |  |  |  |
| Saha-91 |  |  |  |  |  |  |  |  |  |  |  |  |  |  |  |  |  |  |  |  |  |  |  |  |  |  |  |  |  |  |  |  |  |  |  |  |  |  |  |
| Saha-92 |  |  |  | K |  |  |  |  |  |  |  |  | Q | E |  |  |  | I |  |  | T |  |  | I |  |  |  | D |  |  | T |  |  |  |  |  |  |  |  |
| Saha-93 |  |  |  |  |  |  |  |  |  |  |  |  | Q | E |  |  |  | I |  |  | T |  |  | I |  |  |  | D |  |  | T |  |  |  |  |  |  |  |  |
| Saha-94 |  |  |  | K |  |  |  |  |  |  |  |  | Q | E |  |  |  |  |  |  | T |  |  | I | Y |  |  | G |  |  | T |  |  |  |  |  |  |  |  |
| Saha-95 |  |  |  | K |  |  |  |  |  |  |  |  | Q | Q |  |  |  |  |  |  |  |  |  | I | Y |  |  | G |  |  | T |  |  |  |  |  |  |  |  |
| Saha-96 |  |  |  | K |  |  |  |  |  |  |  |  |  |  |  |  |  |  |  |  |  |  |  |  |  |  |  |  |  |  |  |  |  |  |  |  |  |  |  |
